# Validating and Adapting the Brief Resilient Coping Scale for Greek Humanitarian Workers

**DOI:** 10.1192/j.eurpsy.2024.699

**Published:** 2024-08-27

**Authors:** M. Bakola, S. Reppas, A. K. Sakaretsanou, K. S. Kitsou, N. Vaitsis, S. Angelakou-Vaitsi, K. Mavridou, A. Veizis, P. Gourzis, E. Jelastopulu

**Affiliations:** ^1^Department of Public Health, School of Medicine, University of Patras, Patras; ^2^Intersos Hellas, Athens; ^3^Department of Psychiatry, University of Patras, Patras, Greece

## Abstract

**Introduction:**

Humanitarian workers (HWs) face significant challenges while providing aid to those in need, often leading to psychological exhaustion and the risk of primary or secondary trauma.

**Objectives:**

Our study aimed to validate and adapt the Greek version of the Brief Resilient Coping Scale (BRCS) for HWs in Greece.

**Methods:**

We conducted a cross-sectional study between September and December 2022. Participants were recruited through a self-administered questionnaire distributed via social media to humanitarian groups. Additionally, the questionnaire was sent via email to these groups’ members, who then forwarded it to their respective networks. The questionnaire included the BRCS, a 4-item measure designed to capture tendencies to cope with stress in a highly adaptive manner. A score of 4-13 points indicates low resilient copers, 14-16 points medium resilient copers and 17-20 points high resilient copers. Cronbach’s alpha was used to assess internal consistency. Confirmatory Factor Analysis (CFA) was employed to evaluate model fit. Adequate or good fit criteria included a χ2 test p-value ≥ 0.05, Root Mean Square Error Approximation (RMSEA) ≤ 0.08, Standardized Root Mean Squared Residual (SRMR) ≤ 0.05, and a Comparative Fit Index (CFI) or Tucker–Lewis Index (TLI) ≤ 0.90. Statistical analyses were performed using STATA and SPSS software.

**Results:**

A total of 151 humanitarian workers (76% females), with a mean age of **39.3**±10.6 years participated in the study. The mean BRCS score was 65.6/100. Participants were categorized as follows: 34.6% as low resilient copers, 38.6% as medium resilient copers, and 26.8% as high resilient copers. Cronbach’s alpha for the BRCS was 0.84, indicating good internal consistency. CFA results supported the one-factor solution proposed by the original researchers, with acceptable global fit indices: Chi-square p-value = 0.303, SRMR = 0.028, RMSEA = 0.036, CFI = 0.991, TLI = 0.974.

**Conclusions:**

The findings of our study show that the Greek version of BRCS is a valid and reliable tool that can be used to evaluate resilient coping among humanitarian workers in Greece.

**Disclosure of Interest:**

None Declared

